# Evaluation of efficacy and safety of vertebroplasty in the treatment of osteoporotic vertebral compression fracture: A protocol for systematic review and meta-analysis

**DOI:** 10.1097/MD.0000000000027408

**Published:** 2021-10-22

**Authors:** Jie Li, Xin Huang, Guo Zhong

**Affiliations:** a Department of Emergency Surgery, Tianyou Hospital Affiliated to Wuhan University of Science & Technology, Wuhan, Hubei, China; b Department of Orthopedics, Union Jiangbei Hospital (the People's Hospital of Caidian), Wuhan, Hubei, China.

**Keywords:** compression fracture, efficacy, kyphoplasty, osteoporosis, vertebroplasty

## Abstract

**Background:**

Osteoporosis is a common condition that affects bones. Osteoporotic vertebral compression fracture (OVCF) is a highly prevalent complication related to osteoporosis. Each year, OVCF affects nearly 700,000 individuals. Nowadays, there are many surgical methods to treat OVCF, and each has distinct advantages. Among them, the efficacy and safeness of vertebroplasty are yet to be established. The present article will systematically ascertain the effectiveness and security of using vertebroplasty to treat OVCF.

**Methods:**

All articles related to the research topic published from establishment to July 2021 will be retrieved from the following e-databases Medline, Cochrane library, PubMed database, China National of Knowledge Infrastructure, Web of Science, Embase database, and WanFang database. The collected articles will be related to the use of vertebroplasty to treat OVCF. All included articles will adhere to the standards, and will be screened by 2 independent authors. After extracting the data in the article, Review Manager 5.3 software will be used for data analysis.

**Results:**

This study will assess the efficacy and safety of using vertebroplasty to treat OVCF by pooling the results of each study.

**Conclusion:**

Our findings will present strong evidence to help determine whether vertebroplasty therapy is efficient and safe for OVCF patients.

## 1 Introduction

Osteoporosis causes many complications, among them, osteoporotic vertebral compression fracture (OVCF) is most prevalent. It affects almost 700,000 individuals annually.^[1]^ Osteoporosis weakens bones through bone degradation or decreased bone density, increasing the risk of bone fractures. Generally, osteoporotic fractures occur in the spine. In most cases, there are no symptoms until a fracture occurs. Due to lesser bone strength, diminished bone mass, and higher bone fragility, even minor injuries can cause fragility fractures. Most of these fractures are complete fractures, and OVCF is the most common type of fracture.^[2]^ Moreover, the bone healing process slows down after the fracture and surgical treatment becomes more difficult. As a result, the clinical efficacy is reduced and the risk of the fracture recurring increases significantly. The patients’ quality of life is affected. In worst cases, there is a higher likelihood of disability and mortality. A large number of retrospective studies have found that the mortality rate of women aged over 65 years with vertebral compression fractures is 23% more compared to the control group of the same age, and it increases with the increase in the number of vertebral fractures.^[3]^

The existing treatment methods for OVCF include conservative treatment and surgical treatment. Conservative treatments include bed rest, medication analgesia, and external brace fixation. However, conservative treatment cannot rectify spinal deformities. Besides, patients often suffer low back pain for longer periods. Studies do not show an association between the treatment time and the curative effect of each method in conservative treatment, such as the association between fracture healing and periods in bed. Regardless of the treatment type, it needs to be combined with anti-osteoporosis treatment to fundamentally increase bone mass and bone strength to reduce the incidence of re-fracture.^[4]^ Minimally invasive surgery and open surgery are the 2 main types of surgical treatments. Up to now, open surgeries are mostly used to treat patients with nerve and spinal cord compression and structural imbalance. Osteoporosis often leads to failure of internal fixation. Open surgery is traumatic and most patients are elderly. Before the surgery, an assessment should examine the cardiopulmonary function and the tolerance of the surgery. Generally, a bone density examination is performed to assess the severity of osteoporosis. Bone cement reinforcement is often required when internal fixation is implanted. Minimally invasive surgery is more mature and primarily encompasses percutaneous vertebroplasty and percutaneous kyphoplasty, both belong to vertebroplasty. Minimally invasive surgery can achieve stable fractures. The goal of minimally invasive surgery is to restore the mechanical strength of the vertebral body and prevent further compression of the vertebral body. Consequently, it alleviates the patient's pain allowing the patient to resume normal activities. Admittedly, there is no research to prove that vertebroplasty is more effective than conservative treatment for treating OVCF. However, through clinical experience, we believe that early minimally invasive surgery is the best method for OVCF treatment.^[5]^ Currently, minimally invasive surgery and surgical treatment remain controversial, and the scarcity of generalized results to evaluate the effectivity of the 2 methods could be 1 reason for the controversy regarding surgical treatment. A consensus on the outcome parameters could elevate the relevance of the research and facilitate extensive comparative analysis.

## 2 Materials and methods

The study was registered on the OSF (http://osf.io/). If any changes to this agreement are required, they will be recorded on the OSF platform. Since this study does not involve any human subjects, it does not need an approval from the institutional review board.

### 2.1 Information sources

This study will be based on the PRISMA System Evaluation Guide.^[6]^ Search of related meta-analysis and original literature will be conducted in Medline, Cochrane library, PubMed database, China National of Knowledge Infrastructure, Web of Science, Embase database, and WanFang database. The time frame is from the establishment of the database to July 2021.

### 2.2 Search strategy

Search using a combination of the following key∗∗words: vertebroplasty, kyphoplasty, compression fracture, osteoporosis, and osteoporosis. The study is determined by 2 independent reviewers. When there is uncertainty about qualifications, the third reviewers will be consulted, and the disagreement will be resolved through consensus.

### 2.3 Eligibility criteria

#### 2.3.1 Inclusion criteria

(1) All eligible literature should only include randomized controlled trials including patients with OVCF;
(2) The patients should satisfy the clear diagnostic criteria of OVCF;
(3) Intervention measures: the experimental group was treated with vertebroplasty; the control group received conservative treatment or conventional surgery;

#### 2.3.2 Literature exclusion criteria

(1) Non-randomized trials, comments, letters, case reports, editorials, litigation procedures, and personal communications;
(2) Animal research;
(3) Spondylarthritis, medications, traumatic osteoporosis, and exercise therapy;
(4) Non-English and -Chinese articles.

### 2.4 Outcome

Health-related quality of life, operation time, surgery pain, postoperative lying-in bed time, hospital stay, and adverse events, are the measured outcomes. A Visual Analogue Scale or Numeric Rating Scale presents a simple and commonly used instrument to measure pain.^[7,8]^

Several questionnaires can be used to record health-related quality of life, these include, the Short Form 36 General Health Survey,^[9]^ the Quality of Life Questionnaire of the European Foundation for Osteoporosis,^[10]^ the Assessment of Quality of Life^[11]^ questionnaire, and Study of Osteoporotic Fractures-Activities of Daily Living.^[12]^ The Roland-Morris Disability Questionnaire is commonly adopted to examine health when there is pain in the lower back.^[13]^ In 1976, The Oswestry Disability Index was devised in a specialized referral clinic for patients suffering from chronic pain in the lower back region.^[14]^

### 2.5 Study selection and data extraction

A pair of independent researchers will screen the retrieved studies as per the inclusion and exclusion criteria. The screening process will involve importing the retrieved documents into the Endnote X7 document management software to remove duplicate articles. Afterwards, the title and abstract will be scrutinized in each article to further exclude irrelevant studies that fail to satisfy the inclusion criteria. After the preliminary screening, the reviewers will read and analyze the full text of each study to ensure they satisfy the inclusion criteria. After confirming the studies to be included, the next phase is the extraction stage. All disagreements are resolved through discussion or submission to a third party for consultation. The extraction of data will primarily include author, publication time, country, follow-up time, intervention measures, number of cases, and outcome indicators.

### 2.6 Quality assessment

The evaluation of the literature quality will be conducted based on the bias risk assessment tool advocated by the Cochrane Collaboration. The evaluation content includes random order generation, allocation and hiding of random schemes, blinding research subjects and intervention providers, completeness of outcome index data, and selection results of sexual reporting, and other sources of bias. Each item will be evaluated under “low risk of bias”, “unclear risk of bias”, and “high risk of bias”. The quality evaluation will be conducted independently by 2 researchers and cross-checked to discuss and resolve any differences.

### 2.7 Statistical analysis

Review Manager 5.3 will be used to assess the quality of the selected literature and draw a bias analysis chart. STATA15.0 software will be employed to conduct the meta-analysis. The results after analyzing with STATA15.0 are presented with effect size and 95% confidence interval (CI). For continuous data, the effect size is expressed by the mean differences or standardized mean differences and 95% CI. For dichotomous outcomes, the results will be presented as the relative risk with 95% CIs. The heterogeneity assessment will use a chi-squared statistic and I^2^ statistic included in the forest plot. Accordingly, a heterogeneity level exceeding 50% will be considered as substantial, in which case, the random-effects model will be used for analysis. Else, the fixed-effects model will be used.^[15,16]^ A sensitivity analysis will also be conducted to assess the robustness of the findings if there is sufficient data. If there is more than 10 studies, the potential publication bias will be examined using a Funnel plot.

## 3 Discussion

Reportedly, the efficiency and safeness of vertebroplasty to treat OVCF indicate controversial results. Besides, no systematic review has systematically evaluated the clinical therapeutic effects of vertebroplasty as a treatment method for OVCF. Accordingly, this systematic review will evaluate the efficiency and safeness of using vertebroplasty to treat OVCF. Our findings could present significant evidence for clinicians and practitioners to make informed clinical decisions to enhance the life quality of those suffering from OVCF.

## Author contributions

**Conceptualization:** Jie Li, Xin Huang, Guo Zhong.

**Data curation:** Jie Li, Xin Huang, Guo Zhong.

**Formal analysis:** Xin Huang, Guo Zhong.

**Funding acquisition:** Jie Li, Guo Zhong.

**Investigation:** Xin Huang.

**Methodology:** Xin Huang.

**Project administration:** Jie Li, Xin Huang.

**Resources:** Guo Zhong.

**Software:** Xin Huang.

**Supervision:** Jie Li, Guo Zhong.

**Validation:** Jie Li, Xin Huang.

**Visualization:** Guo Zhong.

**Writing – original draft:** Jie Li.

**Writing – review & editing:** Xin Huang, Guo Zhong.
